# Microbial and Transcriptomic Profiling Reveals Diet-Related Alterations of Metabolism in Metabolic Disordered Mice

**DOI:** 10.3389/fnut.2022.923377

**Published:** 2022-07-19

**Authors:** Weize Zhu, Ying Hong, Yue Li, Yan Li, Jing Zhong, Xiaofang He, Ningning Zheng, Lili Sheng, Houkai Li

**Affiliations:** ^1^School of Pharmacy, Shanghai University of Traditional Chinese Medicine, Shanghai, China; ^2^Department of Endocrinology, Shanghai Fifth People's Hospital, Shanghai Medical School, Fudan University, Shanghai, China; ^3^Huzhou Key Laboratory of Molecular Medicine, Huzhou Central Hospital, Huzhou, China

**Keywords:** metabolic syndrome, high-energy diet, gut microbiota, metabolism, transcriptomic profiling

## Abstract

Metabolic disorders are the prelude of metabolic diseases, which are mainly due to the high-energy intake and genetic contribution. High-fat diet (HFD) or high-sucrose diet is widely used for inducing metabolic disorders characterized by increased body weight, insulin resistance, hepatic steatosis, and alteration of gut microbiome. However, the triangle relationship among diets, gut microbiome, and host metabolism is poorly understood. In our study, we investigated the dynamic changes in gut microbiota, and host metabolism in mice that were fed with either chow diet, HFD, or chow diet with 30% sucrose in drinking water (HSD) for continued 12 weeks. The gut microbiota was analyzed with 16S rDNA sequencing on feces. Hepatic gene expression profile was tested with transcriptomics analysis on liver tissue. The host metabolism was evaluated by measuring body weight, insulin sensitivity, serum lipids, and expression of proteins involved in lipid metabolism of liver. The results showed that HFD feeding affected body weight, insulin resistance, and hepatic steatosis more significantly than HSD feeding. 16S rRNA gene sequencing showed that HFD rapidly and steadily suppressed species richness, altered microbiota structure and function, and increased the abundance of bacteria responsible for fatty acid metabolism and inflammatory signaling. In contrast, HSD had minor impact on the overall bacteria structure or function but activated microbial bile acid biosynthesis. Fecal microbiota transplantation suggested that some metabolic changes induced by HFD or HSD feeding were transferrable, especially in the weight of white adipose tissue and hepatic triglyceride level that were consistent with the phenotypes in donor mice. Moreover, transcriptomic results showed that HFD feeding significantly inhibited fatty acid degradation and increase inflammation, while HSD increased hepatic *de novo* lipogenesis and inhibited primary bile acid synthesis alternative pathway. In general, our study revealed the dynamic and diversified impacts of HFD and HSD on gut microbiota and host metabolism.

## Introduction

Metabolic disorder mainly includes dysregulation of glucose and lipid metabolism, insulin resistance, and inflammation, which is predominantly due to the excessive energy intake, in addition to genetic susceptibility (1, 2). Metabolic disorder is the prelude of metabolic diseases consisting of obesity, non-alcoholic fatty liver disease (NAFLD), type 2 diabetes (T2DM), hyperlipidemia, and metabolic syndrome (3–5). It is well known that the prevalence of Western diet is the key factor contributing to the development of metabolic disorders (6–9).

The commensal gut microbiome is regarded as a “metabolic organ” for host (10), which not only takes part in intestinal nutrients absorption (11, 12), but also regulates host metabolism by producing microbial metabolites like short-chain fatty acids (acetate, propionate, butyrate, etc.) (13–15), or secreting peptides like GLP-1 modulating glucose homeostasis (16). On the contrary, bacteria-derived lipopolysaccharide (LPS) induces metabolic endotoxemia resulting to development of metabolic diseases (17–21). Increasing evidence has demonstrated the involvement of gut microbiome in metabolic diseases, which are characterized by dramatic changes in gut microbiota in both structure and function (22, 23). Although the causative roles of gut microbiome in metabolic diseases have been well-evidenced, the exact relationship among gut microbiome, diets, and metabolic disorders is still elusive.

High-energy diets are widely used in experimental studies to induce animal models with metabolic disorders, including high-fat diet (HFD) and/or high-sugar (sucrose/ fructose/glucose) diet (HSD) (24–26). The diet-induced animal models with metabolic disorders are not only characterized by typical phenotypes that are present in human patients (26, 27), but also show dramatic changes in gut microbiome (28, 29). Yang et al. (30) reported the significant increases in pathogenic bacteria *Alistipessp.Marseille-P5997* and *Alistipessp.5CPEGH6* while reduced abundance of *Parabacteroides distasonis* in HFD-fed mice, accompanied by impaired gut barrier function. Wang et al. (31) found that HFD-fed mice showed significantly altered *Blautia, Desulfovibrio*, and Lachnospiraceae_NK4A136_group abundance, whereas decreased abundance of *Desulfovibrio vulgaris* was also observed in HFD-fed mice, and supplement of *Desulfovibrio vulgaris* improved HFD-induced hepatic steatosis (32). Meanwhile, the impacts on gut microbiota and gut permeability were investigated in mice that were fed with high glucose or fructose diets, which showed that high glucose or fructose feeding resulted in poor gut microbial diversity, characterized by a lower proportion of Bacteroidetes and increased Proteobacteria, as well as altered expression of tight junction proteins (33). As a result, although the dietary impacts on gut microbiota were well-established (34–36), the exact relationship between diet-induced alteration of gut microbiota and host metabolism remains unclear.

In this study, we systemically evaluated the dynamic impacts of a 12-week HFD and high-sucrose feeding on gut microbiota and host metabolism in mice by measuring the body weight gain, serum lipids, hepatic steatosis, glucose tolerance and insulin sensitivity, composition of gut microbiota, and hepatic gene expression profiles. Our results showed that HFD feeding induced more significant changes in insulin resistance, hepatic steatosis, gut microbiota, and gene expression profile of liver than HSD feeding in the context of comparable amount of energy intake. Moreover, the gut microbiota from HFD and HSD feeding mice induced different impacts on metabolism. Taken together, our results revealed the dynamic and diversified impacts of HFD and HSD feeding on gut microbiota and host metabolism.

## Materials and Methods

### Mice

All animals were purchased from Shanghai SLAC Laboratory Animal Co., Ltd and bred at the experimental animal center, Shanghai University of Traditional Chinese Medicine. They were housed in a regulated barrier system facility at 23–24°C with 60 ± 10% relative humidity and a 12:12-h light/dark cycle under specific-pathogen-free (SPF) grade. All experimental procedures were approved by the Animal Experiment Institution of Shanghai University of Traditional Chinese Medicine.

### Time-Course Animal Experiment

After 1-week accommodation, 105 4-week-old male C57BL/6 mice were randomly divided into three groups and were fed with chow diet and normal drinking water (Con, *n* = 30), chow diet and 30% sucrose in drinking water (HSD, *n* = 30, #V900116, Merck, Germany), or high-fat diet and normal drinking water (HFD, *n* = 45, 60% fat, #D12492, Research Diet, USA, Table 1). Ten mice from Con and HSD groups or 15 mice from HFD group were killed with 1% pentobarbital sodium solution intraperitoneally at the 4th, 8th, and 12th week, respectively. Tissue samples were collected, weighted, and immediately frozen in liquid nitrogen and stored at−80°C for further analysis. Part of liver tissue and epididymal fat tissue were fixed with 10% neutral formalin for subsequent HE staining. Tissue index = tissue weight (g)/ body weight (g).

**Table 1:** Caloric information of diet.

**Caloric information**	**Chow diet**	**High-sucrose diet**	**High-fat diet**
Protein:	22.8% Kcal	22.8% Kcal	20.0% Kcal
Fat:	13.8% Kcal	13.8% Kcal	60.0% Kcal
Carbohydrate:	63.4% Kcal	63.4% Kcal	20.0% Kcal
Energy density in diet:	3.65 Kcal/g	3.65 Kcal/g	5.21 Kcal/g
Energy density in drinking water:	0.00 Kcal/ml	1.31 Kcal/ml	0.00 Kcal/ml

### Fecal Microbiota Transplantation Animal Experiment

In the FMT experiment, 4-week-old male C57BL/6 mice were divided into five groups: Con, R-HSD, R-HFD, D-HSD, and D-HFD. Con group were fed with chow diet and normal drinking water (Con, *n* = 6). D-HSD were fed with chow diet and 30% sucrose in drinking water (D-HSD, *n* = 6), and D-HFD were fed with high-fat diet and normal drinking water (D-HFD, *n* = 6) during the entire experiment. After 4 weeks of HSD or HFD feeding, fresh fecal sample was continuously collected while study continued and fed to recipient groups. R-HSD and R-HFD groups were fed with chow diet and normal drinking water and received antibiotics for 1 week before FMT to deplete the gut microbiota (oral gavage of 10 g/L Metronidazole, 5 g/L Vancomycin, and 10 g/L Neomycin at the dose of 0.1 mL/10 g body weight, 1 g/L Ampicillin in drinking water, *n* = 6 per group). Oral gavage of the bacterial suspension from D-HSD or D-HFD groups started from Week 4 and continued for 4 weeks. The bacterial suspension procedure is as follows: Freshly collected feces were diluted in PBS at a ratio of 50 mg feces/ml PBS, homogenized, and filtered with a germfree sieve (0.2 mm, Thermo, USA), and the R-HSD and R-HFD group mice were administered 200 μl of the fecal suspension by oral gavage once every day for 4 weeks.

### Histological Evaluation on the Degree of Hepatic Steatosis

Liver tissues were fixed with 10% formalin for 24 h, embedded in paraffin, and then stained with hematoxylin–eosin staining (H&E) using a standard protocol. Hematoxylin staining was used for nuclear counterstaining (blue), and eosin was used to stain the cytoplasm red. The degree of hepatic steatosis was evaluated according to previous publication in a blinded way (37). The criteria for scoring include grade 0, no steatosis; grade 1, steatosis involved <25%; grade 2, steatosis involved between 26 and 50 %; grade 3, steatosis involved between 51 and 75 %; and grade 4, steatosis involved >75%.

### Serum Biochemistry Index Test

Mice were fasted for 12 h, and blood sample was collected before mice been killed, left undisturbed at room temperature for 1 h, and then centrifugated at 4°C, 4,000 rpm for 10 min to separate serum. Serum TC (#A111-1-1, Nanjing Jiancheng, China), TG (#A110-1-1, Nanjing Jiancheng, China), HDL-C (#A112-1-1, Nanjing Jiancheng, China), LDL-C (#A113-1-1, Nanjing Jiancheng, China), ALT (#C009-2-1, Nanjing Jiancheng, China), NEFA (#A042-2-1, Nanjing Jiancheng, China), and insulin (#EZRMI-13K, Merck, Germany) were tested according to the instruction manual.

### Liver Triglycerides Examination

Hepatic lipids were extracted according to the optimized Folch method (38). Briefly, 12.5 mg of liver tissue was homogenized in 500 μl of chloroform: methanol (2:1, v: v) and centrifuged briefly. Then, tissues were grinded at 60 Hz for 1 min, vortexed for 1 min, and centrifugated at 4°C, 4,000 rpm for 10 min. About 10 μl (60 μl for TC test) supernatant was collected and dried at room temperature. The residue was resuspended with 10 μl ultra-pure water, and the TG and TC levels were measured with the instruction manual.

### Glucose Tolerance Test and Insulin Tolerance Test

GTT was conducted in mice after overnight fasting. Then, mice were injected intraperitoneally with 10% (g/v) glucose solution at a dosage of 1 g/kg body weight, and then, blood glucose levels were measured using glucose meter (ACCU-CHEK Performa, Germany) at 0, 15th, 30th, 60th, 90th, and 120th min. ITT was performed in fed mice in which all of the mice were intraperitoneally injected with human insulin (0.75 units/kg body weight, *Novo* Nordisk) according to previous test, and then, blood glucose levels were measured essentially as earlier described.

### 16S rRNA Gene Sequencing and Analysis

The genomic DNA extraction and the processing and quality control of raw sequencing data were performed according to the previous study (32). Briefly, the genomic DNA was extracted by QIAamp DNA Mini Kit (#51304, QIAGEN, Germany). Then, the qualified DNA samples were amplified by universal primers of 16S rDNA V3–V4 region (338F and 806R) (39) and sequenced by Illumina MiSeq PE300 system (Illumina, USA). After pretreatment by Fastp (40)and FLASH (41), sequences were divided into operational taxonomic units (OTUs) which have high similarity (≥97%) by UPARSE (42) and removed chimeric sequences by UCHIME (43) based on Silva 16S rRNA database (SSU123) (44). The taxonomy of each sequence was analyzed by RDP classifier algorithm (45) with confidence threshold of 70%. Alpha diversity of each sample was assessed by Mothur (46), including Chao, Ace, and Sob index for bacterial richness analysis, with Simpson and Shannon index for bacterial richness and evenness analysis. The principal coordinate analysis (PCoA) was conducted to reflect community similarity of gut microbiota in each group, distance algorithm was weighted by UniFrac (47), and PERMANOVA analyses assessed by QIIME (48) were conducted to evaluate the significant differences among groups. Relative abundances were calculated by dividing the absolute abundances per phylum, family, or genus by the total sequence count per sample. To predict potential microbial functional, OTUs from each microbial sample were predicted using the PICRUSt (49). The Spearman rank coefficient was used to assess correlations between genus level and phenotypes.

### Transcriptomics Array

The RNA extraction and the processing and quality control of raw sequencing data were performed according to the previous study (32). Briefly, total RNA was extracted by TRIzol (#15596018, Invitrogen, USA), and quality was determined. Then, established RNA library, reverse synthesize cDNA by kits, and the cDNA, as a probe, were immobilized on the chip, labeled with fluorescent molecules, and then hybridized with the chip, and the changes in gene expression levels can be detected by analyzing the fluorescence intensity of the hybridization of the probe. The principal component analysis (PCA) was performed to compare gene similarities of hepatic tissues in each group. KEGG pathway enrichment analysis was performed by the DAVID Bioinformatics Database (http://david.abcc.ncifcrf.gov/).

### Statistical Analysis

Data are shown as means ± s.e.m unless otherwise noted. Statistical significance of body weight and biochemistry index was determined with the unpaired two-tailed Student's *t*-test. The statistical significance of hepatic steatosis scores was evaluated with non-parametric Kruskal–Wallis test followed by the Mann–Whitney *U*-test. *P* < 0.05 was considered statistically significant.

## Results

### High-Fat Diet and High-Sucrose Intake Caused Divergent Changes in Metabolism in Mice

**Figure 1:**
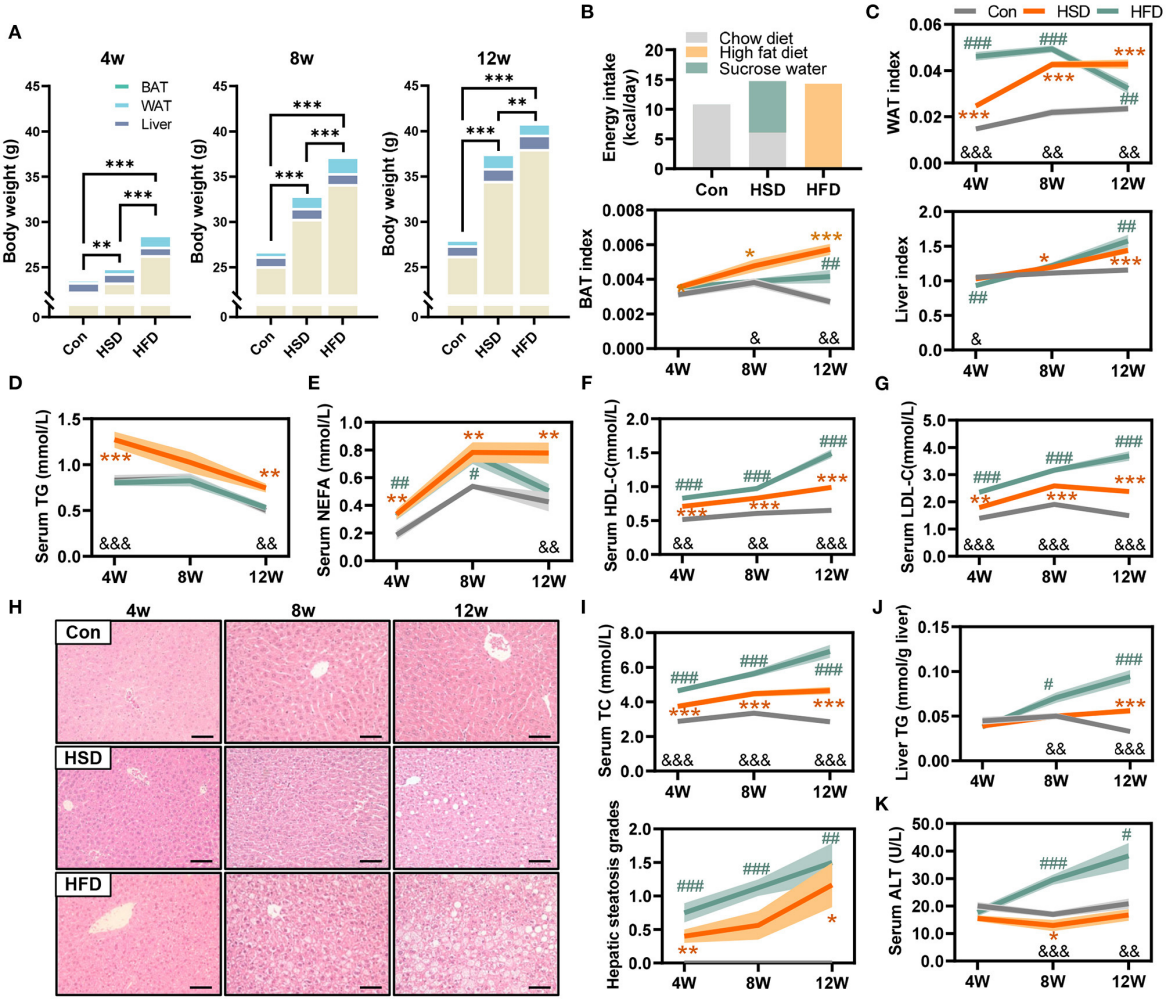
High-fat diet and high-sucrose intake caused divergent changes in metabolism in mice. Male C57BL/6J mice (4 weeks old) were, respectively, treated with chow diet (Con), high-sucrose diet (HSD), or high-fat diet (HFD) for 4, 8, and 12 weeks. **(A)** Body weight and tissue distribution, including liver, white adipose tissue (WAT), and brown adipose tissue (BAT) (g). **(B)** Average energy intake per mouse (kcal/day). **(C)** WAT, BAT, and liver index (index=tissue weight/body weight). **(D)** Serum triglyceride (TG) level (mmol/L). **(E)** Serum non-esterified fatty acid (NEFA) level (mmol/L). **(F)** Serum high-density lipoprotein cholesterol (HDL-C) level (mmol/L). **(G)** Serum low-density lipoprotein cholesterol (LDL-C) level (mmol/L). **(H)** Representative photomicrographs of liver tissue with H&E staining (magnification, ×200, 50 μm), with corresponding hepatic steatosis scores. **(I)** Serum total cholesterol (TC) level (mmol/L). **(J)** Hepatic TG level (mmol/g liver). **(K)** Serum ALT level (U/L). Data are represented as the mean ± SEM. *N* = 10–15; *p < 0.05, **p < 0.01, ***p < 0.001 (HSD *vs*. Con, except plot A); #*p* < 0.05, ##*p* < 0.01, ###*p* < 0.001 (HFD *vs*. Con); &*p* < 0.05, &&*p* < 0.01, &&&*p* < 0.001 (HFD *vs*. HSD).

To test the effect of different high-energy diets on the overall metabolism, a series of indicators of metabolic disorder was measured in high-fat and high-sucrose diet-fed mice with different intervention durations. Mice were fed with chow diet, high-fat diet (HFD, 60% fat), or high-sucrose diet (HSD, 30% sucrose in water) for 4, 8, and 12 weeks. First, both HFD and HSD led to time-dependent increases in body weight over 12 weeks with higher body weight in HFD-fed mice, though both groups had the same energy intake (Figures 1A,B). The weight and ratio of white adipose tissue (WAT), brown adipose tissue (BAT), and liver were significantly increased in HFD and HSD-fed mice, with higher WAT and BAT weight with ratio in HSD group after 12-week intervention (Figures 1A,C). Both diets elevated serum lipid. However, serum triglycerides (TG) were much higher in HSD-fed mice than HFD-fed mice at all three studied time point, while non-esterified fatty acid (NEFA) was higher in HSD-fed mice than HFD-fed mice after 12-week intervention (Figures 1D,E). In contrast, serum high-density lipoprotein cholesterol (HDL-C), low-density lipoprotein cholesterol (LDL-C), and total cholesterol (TC) were higher in HFD group than HSD group at all-time points (Figures 1F–I). In addition, HFD induced more severe fat accumulation in the liver, which was revealed by higher hepatic steatosis grade and liver TG (Figures 1H,J). Moreover, serum alanine aminotransferase (ALT) was only elevated in HFD group (Figure 1K). These findings suggested that both HFD and HSD can cause metabolic disorders with different extends, and compared to HSD, HFD has a more significant effect on inducing fatty liver and liver injury.

**Figure 2:**
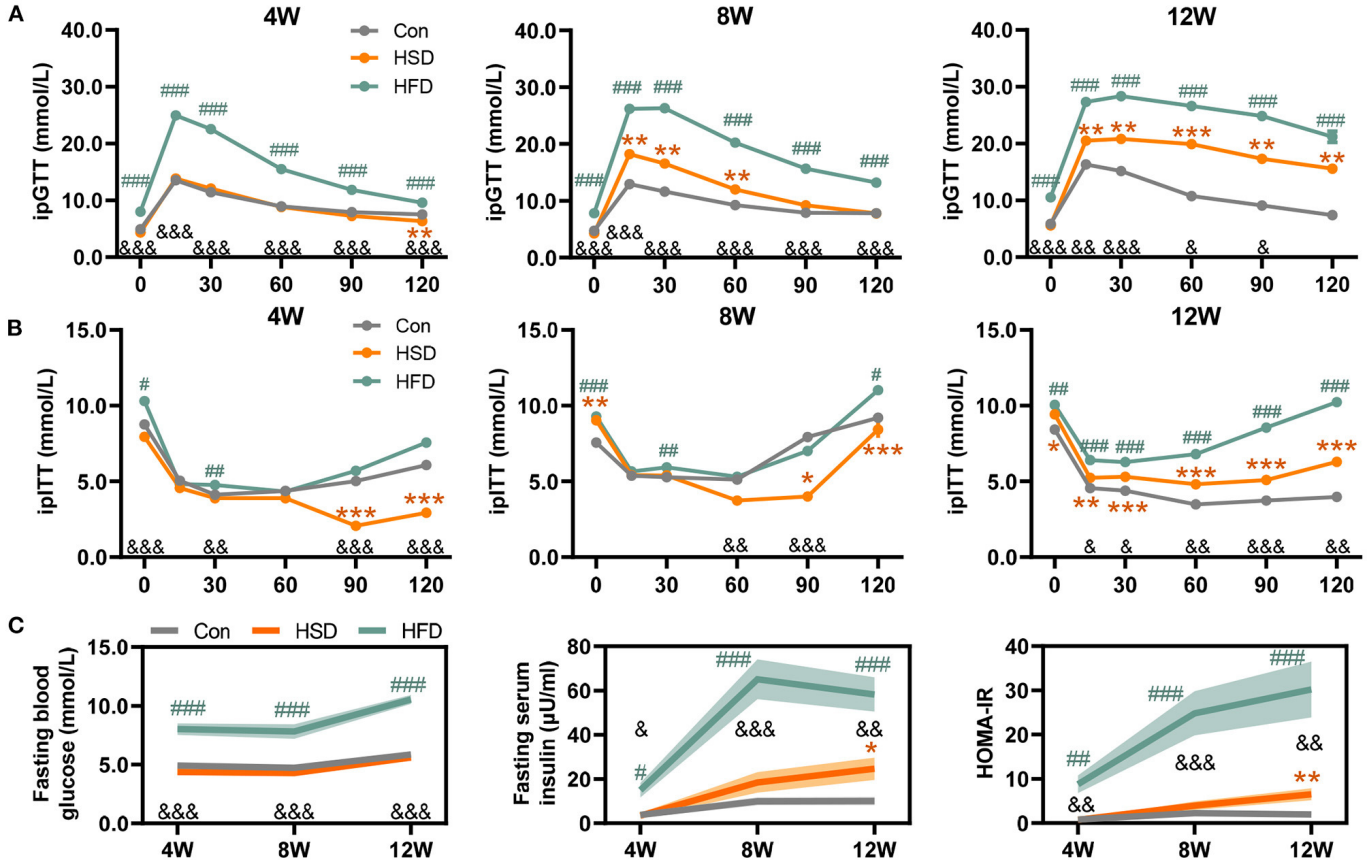
High-fat or high-sugar diet-induced the impaired glucose tolerance in mice. **(A)** Glucose tolerance test (GTT) was performed at 1 week before 3 time points (mmol/L). **(B)** Insulin tolerance test (ITT) was performed 3–4 days after each GTT test (mmol/L). **(C)** Fasting blood glucose level (mmol/L), fasting serum insulin level (μU/ml), and HOMA-IR (fasting blood glucose level * fasting serum insulin level / 22.5). Data are represented as the mean ± SEM. *n* = 7-15; *p < 0.05, **p < 0.01, ***p < 0.001 (HSD *vs*. Con); #*p* < 0.05, ##*p* < 0.01, ###*p* < 0.001 (HFD *vs*. Con); &*p* < 0.05, &&*p* < 0.01, &&&*p* < 0.001 (HFD *vs*. HSD).

To further explore the effect of different dietary intakes on glucose metabolism, we conducted glucose tolerance tests (GTT) and insulin tolerance tests (ITT) after 4-, 8-, and 12-week intervention. The result showed HFD induced more severe glucose intolerance than HSD. HFD induced glucose intolerance as early as 4 weeks, while HSD started to induce glucose intolerance only after 8 weeks (Figure 2A). The results of ITT test also show that the decrease in insulin sensitivity occurs after 12-week intervention in both HFD and HSD groups (Figure 2B). Meanwhile, we found HFD, but not HSD, could significantly increase fasting blood glucose level, fasting serum insulin level, and HOMA-IR (Figure 2C). These results indicated that HFD and HSD could both induce glucose metabolism disorder, with more severe degree and earlier in HFD group than HSD group.

### High-Fat Diet, but Not High-Sucrose Intake Resulted in Dramatic Alteration of Gut Microbiome

**Figure 3:**
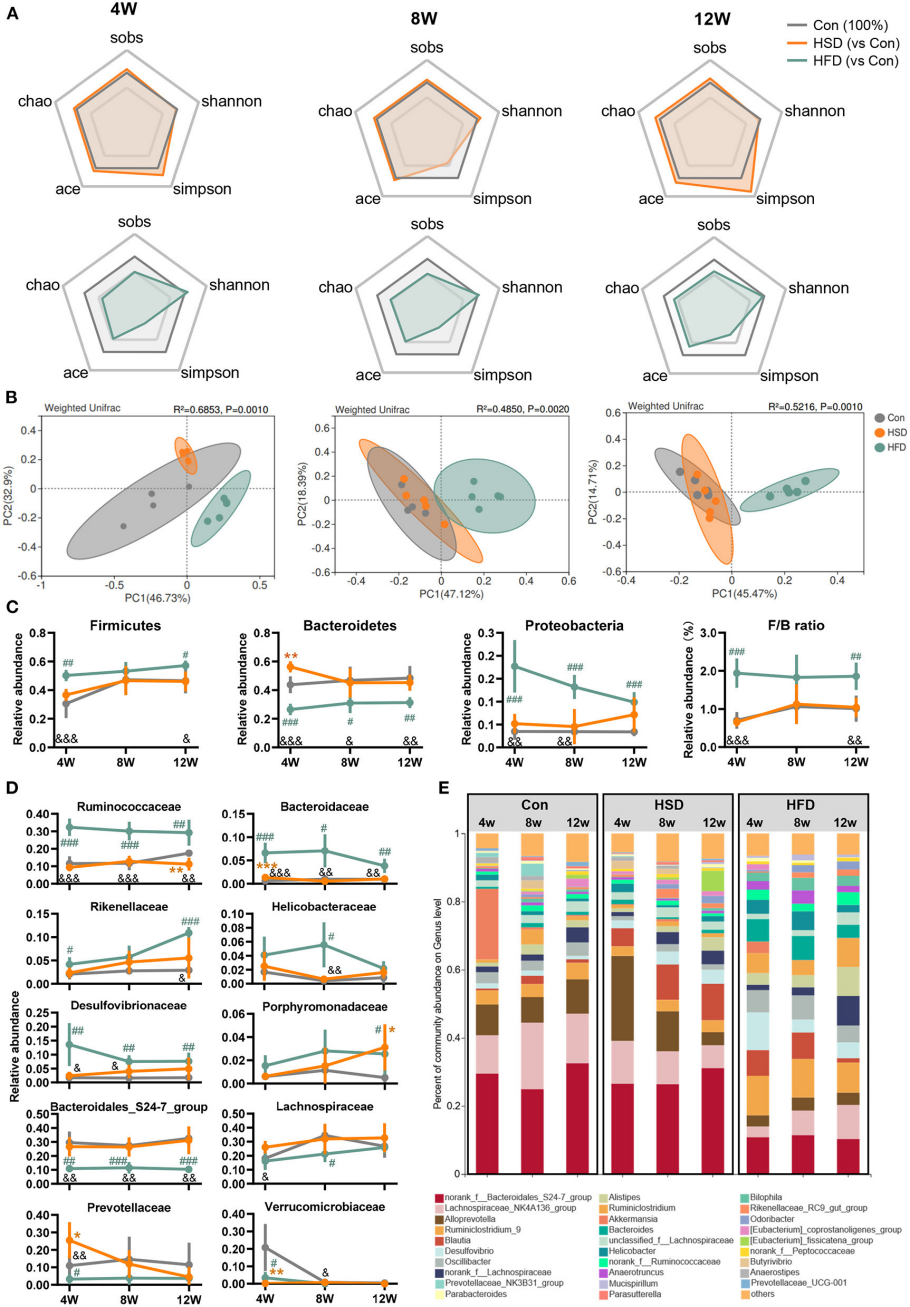
High-fat diet, but not high-sucrose intake resulted in dramatic alteration of gut microbiome. Fecal samples of Con, HSD, and HFD groups at three time points were analyzed with 16S rRNA gene sequencing. **(A)** The relative change in α diversity index, including Sobs, Chao, Ace, Simpson, and Shannon index, normalized with Con group (100%). **(B)** Weighted UniFrac based PCoA analysis among groups with permutational multivariate analysis of variance (PERMANOVA). **(C)** Relative abundance of bacteria at phylum level and Firmicutes/Bacteroidetes (F/B) ratio. **(D)** Relative abundance of top 10 abundant family. **(E)** Bacterial taxonomic profiling at genus level. Data are represented as the mean ± SEM. *n* = 4-5; *p < 0.05, **p < 0.01, ***p < 0.001 (HSD *vs*. Con); #*p* < 0.05, ##*p* < 0.01, ###*p* < 0.001 (HFD *vs*. Con); &*p* < 0.05, &&*p* < 0.01, &&&*p* < 0.001 (HFD *vs*. HSD).

Extensive research has shown that gut dysbiosis is a central initiator of obesity-related diseases including NAFLD, type 2 diabetes, and metabolic syndrome (50, 51). Here, we compared the effects of HFD or HSD on the community structures of the gut flora after 4-, 8-, and 12-week dietary intervention. The compositional alteration of gut microbiota was evaluated based on 16S rRNA gene sequencing. An average of 37,740 ± 504 valid reads was obtained that covered the majority of bacterial diversity. We found that HFD reduced bacterial richness (including Sobs, Chao, and Ace indexes) and Simpson index, with no significant difference in Shannon index at all-time points. However, HSD did not affect bacterial richness and evenness, with the change in Simpson index to a small extent (Figure 3A). The weighted UniFrac principal coordinate analysis (PCoA) also showed that HFD groups were significantly separated from Con and HSD groups on PCoA1, with no separation on Con and HSD groups (Figure 3B). In addition, at the phylum level, HFD increased the relative abundance of Firmicutes and Proteobacteria, but reduced Bacteroidetes, leading to the increase in Firmicutes/Bacteroidetes (F/B) ratio compared to the Con group, with no variation detected between HSD and Con groups (Figure 3C). Interestingly, although the relative abundance of Proteobacteria was elevated in HFD, it was decreased with the increase of intervention time (Figure 3C). We also analyzed the top 10 families which covered 96.09% of total bacteria. Most families showed significant change in HFD group. For instance, HFD increased the abundance of Ruminococcaceae, Bacteroidaceae, Rikenellaceae, Helicobacteraceae, Desulfovibrionaceae, and Porphyromonadaceae, but reduced the abundance of Bacteroidales_S24-7 group, Lachnospiraceae, Prevotellaceae, and Verrucomicrobiaceae (Figure 3D). HSD altered the relative abundance of several family level, with reduced Verrucomicrobiaceae and increased Prevotellaceae and Bacteroidaceae at 4 weeks, as well as reduced Ruminococcaceae and increased Porphyromonadaceae at 12 weeks (Figure 3D). At genus level, HFD increased the abundance of *Ruminiclostridium_9, Blautia, Desulfovibrio, Oscillibacter, Alistipes, Ruminiclostridium*, and *Bacteroides*, but reduced the abundance of *Lachnospiraceae_NK4A136_group, Alloprevotella*, and *Akkermansia*, which aligned with the family-level distinction. Differ from HFD group, HSD increased *Alloprevotella* at 4 and 8 weeks and reduced the abundance of *Ruminiclostridium_9* and *Oscillibacter*, leading to the opposite trend on Ruminococcaceae and Prevotellaceae compare with HFD (Figure 3E).

**Figure 4:**
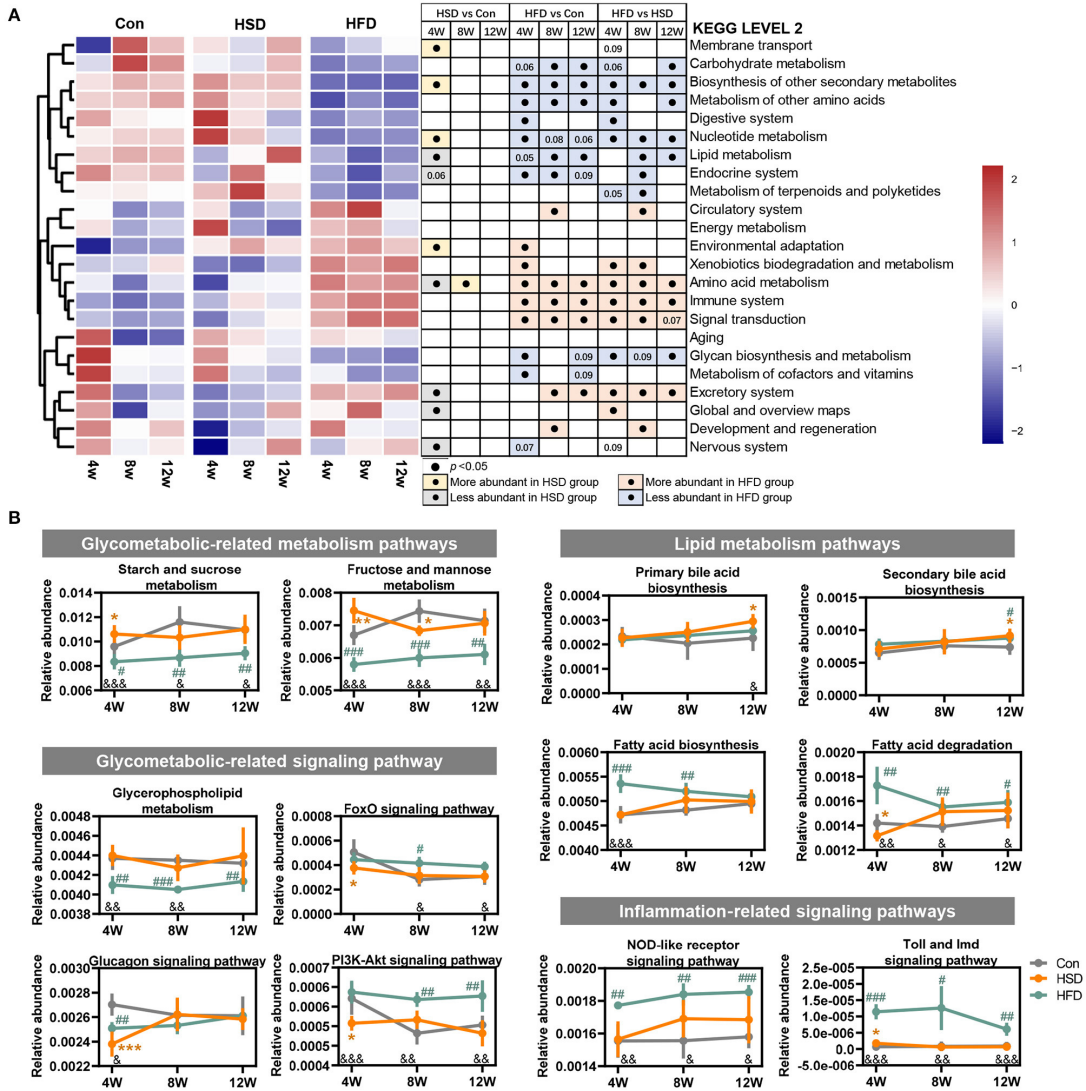
High-fat diet changed the main functions of gut dysbiosis in mice. Microbial functions regulated by HFD or HSD were predicted with PICRUSt2. **(A)** Heatmap summarizing the changes in gut microbial community function at KEGG level 2, following Con, HSD, and HFD group at three time point in mice. Black circle represents the KEGG pathway which was significantly altered relative to Con or HSD groups at same time point (*p* < 0.05). **(B)** Relative abundance of the pathways related to glycolipid metabolism disorder. Data are represented as the mean ± SEM. *n* = 4-5; *p < 0.05, **p < 0.01, ***p < 0.001 (HSD *vs*. Con); ^#^*p* < 0.05, ^*##*^*p* < 0.01, ^*###*^*p* < 0.001 (HFD *vs*. Con); ^&^*p* < 0.05, ^&&^*p* < 0.01, ^&*&&*^*p* < 0.001 (HFD *vs*. HSD).

To assess whether gut microbiota alterations influenced gut microbiota function, PICRUSt2 analysis was performed for functional profile predictions of microbiota based on 16S rRNA gene. We found that the huge impact of HFD on microbiota structure was also accompanied by significant changes in microbial function. Compared with the Con group, HFD-induced bacterial functional changes in KEGG level 2 mainly involved in reduced biosynthesis of secondary metabolites, other amino acids metabolism, nucleotide metabolism, lipid metabolism, endocrine system, and increased amino acid metabolism, immune system, and signal transduction. In contrast, HSD regulated bacterial function only at early stage (Figure 4A). When comparing the effect of HFD with HSD, HFD activated xenobiotic biodegradation and metabolism, amino acid metabolism, immune system signal transduction, and excretory system, while HSD activated carbohydrate metabolism, biosynthesis of other secondary metabolites, metabolism of other amino acids, nucleotide metabolism, and lipid metabolism in most cases. Further functional characterization of the above pathway at KEGG level 3 showed that HFD significant reduced bacterial glucose metabolism including starch and sucrose metabolism, fructose and mannose metabolism, and glycerophospholipid metabolism, with early significant reduction in glucagon signaling and following significant increase in PI3K-Akt signaling. HFD also increased microbial fatty acid metabolism at early stage, including fatty acid biosynthesis and fatty acid degradation, and continuous increased inflammation-relative signaling, including NOD-like receptor signaling pathway and toll and imd signaling pathway (Figure 4B). HSD activated microbial glucose metabolism and reduced insulin regulation signal only at only early stage, but activated primary bile acid biosynthesis and secondary bile acid biosynthesis only at late stage (Figure 4B). The above findings suggested that the impact of HFD on gut microbiota was more robust and sustained than HSD.

### Diet-Dependent Specific Correlation Between Mouse Phenotypes and Gut Microbiota

**Figure 5:**
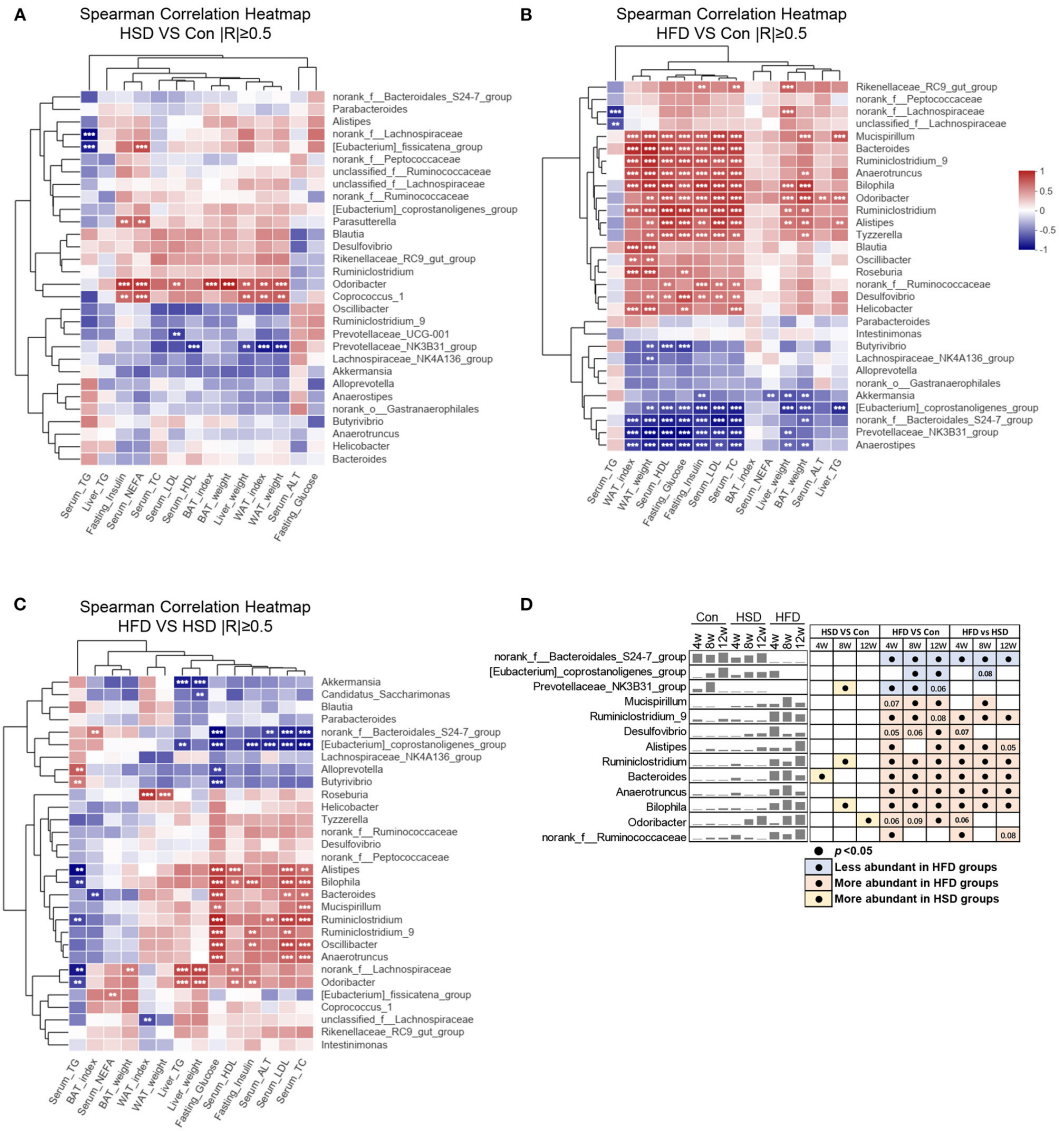
Diet-dependent specific correlation between mouse phenotypes and gut microbiota. Spearman's correlation analysis (|R| ≥ 0.5) between the top 30 abundance genera and metabolic disorder traits (including liver weight, WAT weight, WAT index, BAT weight, BAT index, serum HDL, serum LDL, serum TC, serum TG, serum NEFA, liver TG, serum ALT, fasting glucose, and fasting insulin) among three groups, by a pairwise comparisons, including HSD vs. Con **(A)**, HFD vs. Con **(B)**, and HFD vs. HSD **(C)**. **p < 0.01, ***p < 0.001. **(D)** Mean abundance variation and statistical results of several high phenotypic correlations genus among groups at all-time points. Black circle represents *p* < 0.05.

To explore the relationship between phenotypes and gut microbiota, we performed Spearman's correlation based on different diets. When comparing HSD and Con groups, we noticed *Odoribacter* and *Coprococcus_1* were positively correlated with liver and WAT weight, as well as fasting insulin and serum NEFA level, while *Prevotellaceae_NK3B31_group* was negatively correlated with liver and WAT weight (Figure 5A). When comparing HFD and Con groups, *Odoribacter* and *Prevotellaceae_NK3B31_group* also correlated with most of these phenotypes, which is similar as we found in HSD vs. Con groups. However, more families were either positively or negatively with mouse phenotypes in HFD groups, suggesting HFD-induced dramatic changes in gut microbiota might account for diet-induced metabolic disorder (Figure 5B). Next, we further studied how different gut microbiota compositions in HSD and HFD groups are related to mouse phenotypes. *Norank_f_Bacteroidales_S24-7_group* and *[Eubacterium]_coprostanoligenes_group*, the abundance of which were elevated in HFD than HSD group, were negatively associated with serum TC, LDL, ALT, and fasting glucose level. Some bacterial families that were increased in both HFD vs. Con and HFD vs. HSD groups, such as *Alistipes, Bilophila, Bacteroides, Mucispirillum, Ruminiclostridum, Ruminiclostridum_9, Oscillibacter*, and *Anaerotruncus*, were positively related to serum TC, LDL, ALT, HDL, as well as fasting insulin and glucose level in most cases (Figures 5C,D). These findings suggested that certain bacterial families shifted by HFD might account for the more severe metabolic disorders in HFD-fed mice.

### High-Fat and High-Sucrose Diet-Induced Lipid Metabolic Dysregulation Is Gut Microbiota-Dependent

**Figure 6:**
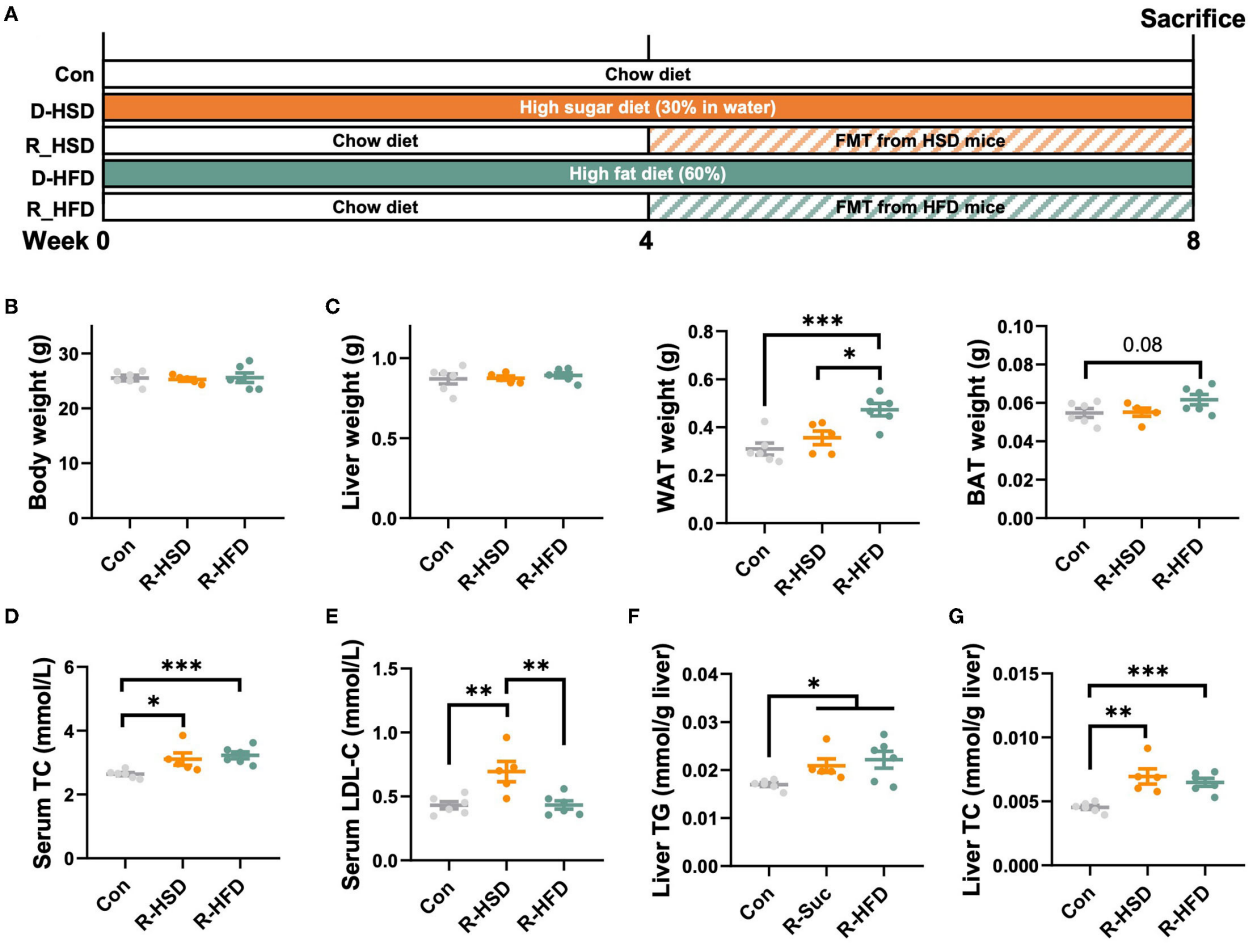
High-fat and high-sucrose diet-induced lipid metabolic dysregulation is gut microbiota-dependent. **(A)** Male C57BL/6J mice (4 weeks old) were, respectively, treated with chow diet (Con), high-sucrose-diet (D-HSD), or high-fat diet (D-HFD) for 4 weeks, and then, fecal bacteria collected from donor mice in each group were pooled and an equal volume was orally transplanted to recipient mice (R-HSD, R-HFD), respectively, at following 4 weeks. Recipient mice were fed with chow diet throughout the experiment. **(B)** Body weight (g). **(C)** Tissue weight, including liver, WAT, and BAT (g). **(D)** Serum TC level (mmol/L). **(E)** Serum LDL-C level (mmol/L). **(F)** Hepatic TG level (mmol/g liver). **(G)** Hepatic TC level (mmol/ g liver). Data are represented as the mean ± SEM. *n* = 5-6; *p < 0.05, **p < 0.01, ***p < 0.001.

To test whether the phenotypic changes in HFD- and HSD-fed mice were due to the modulation of gut microbiota composition and function, a fecal microbiota transplantation (FMT) experiment was performed (Figure 6A). Though no statistical differences were observed in the body weight, liver weight, and BAT weight among R-HFD, R-HSD, and Con mice (Figures 6B,C), R-HSD and R-HFD mice showed similar increase in serum TC, and hepatic TG and TC levels (Figures 6D–G). However, higher increase in WAT weight was observed in R-HFD mice than R-HSD group, while serum LDL levels were higher in R-HSD mice (Figures 6C,E), implying that gut microbiota from HFD or HSD mice induced different impacts on recipient mice. These results suggested that the metabolic influence of HSD and HFD is different, and transferable via gut microbiota at certain extent, highlighting the different contributions to metabolic phenotypes of diet-changed gut microbiota in regulating host metabolism.

### High-Fat Diet and High-Sucrose Intake Differently Altered the Hepatic Transcriptomic Profile

**Figure 7:**
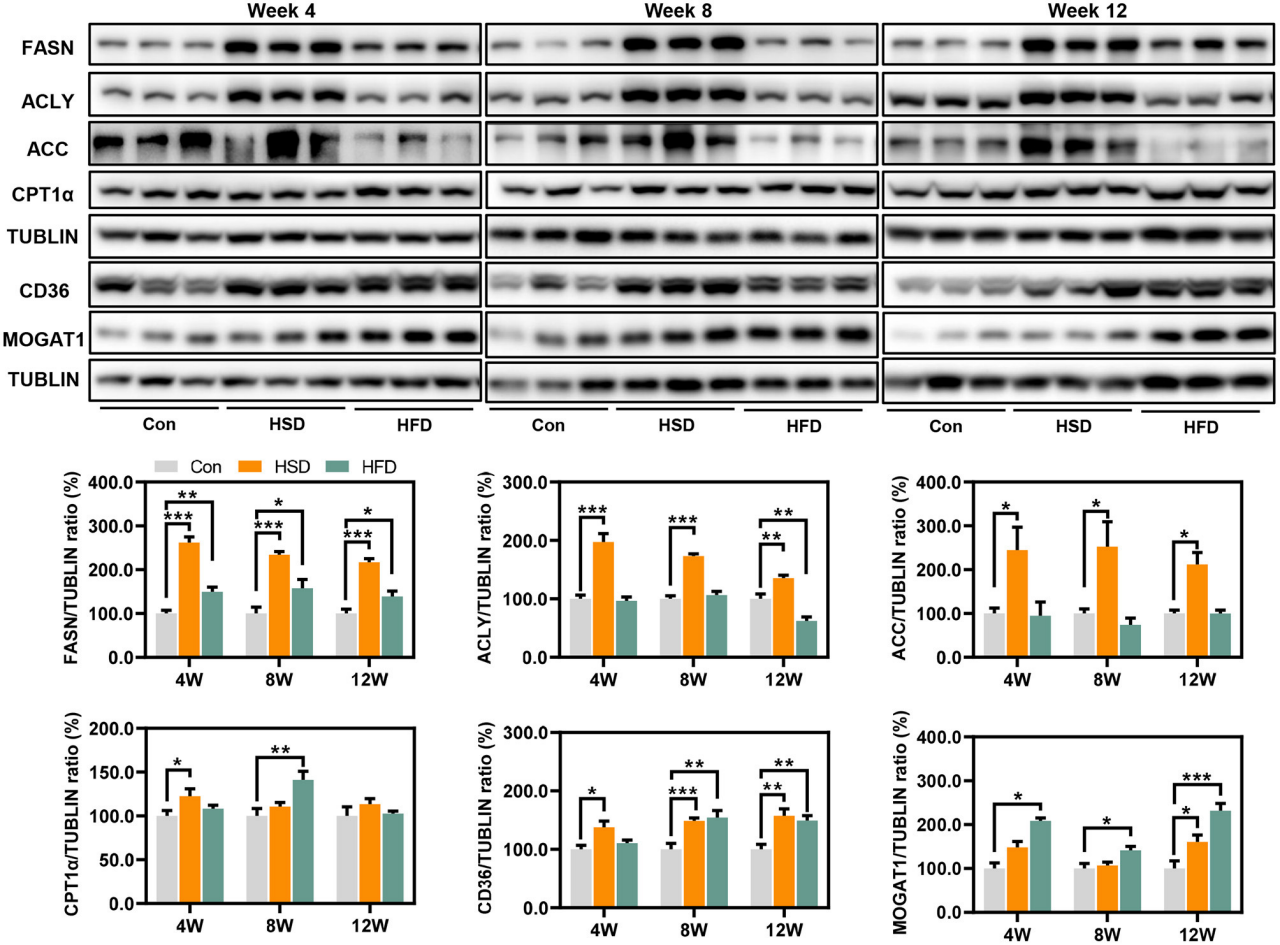
Expression of protein related to hepatic de novo synthesis and metabolism of fatty acid, including FASN, ACLY, ACC, CPT1α, CD36, and MOGAT1 (%). *p < 0.05, **p < 0.01, ***p < 0.001.

To explore the regulation of hepatic lipid metabolism under different energy intake conditions, we tested the expression of protein related to hepatic *de novo* synthesis and metabolism of fatty acid. The result showed that HSD can significantly activate the *de novo* synthesis of endogenous fatty acids [fatty acid synthase (FASN), ATP citrate lyase (ACLY), and acetyl-CoA carboxylase (ACC)] in the liver, although hepatic steatosis was not severe (Figure 7). HSD and HFD could slightly increase the expression of CPT1α, the rate-limiting enzyme for fatty acid β oxidation, at early stage. Meanwhile, HSD and HFD differently upregulated the protein level of fatty acid transporter CD36 and monoacylglycerol acyltransferase (MOGAT1), leading to the increased accumulation of lipid in the liver (Figure 7).

**Figure 8:**
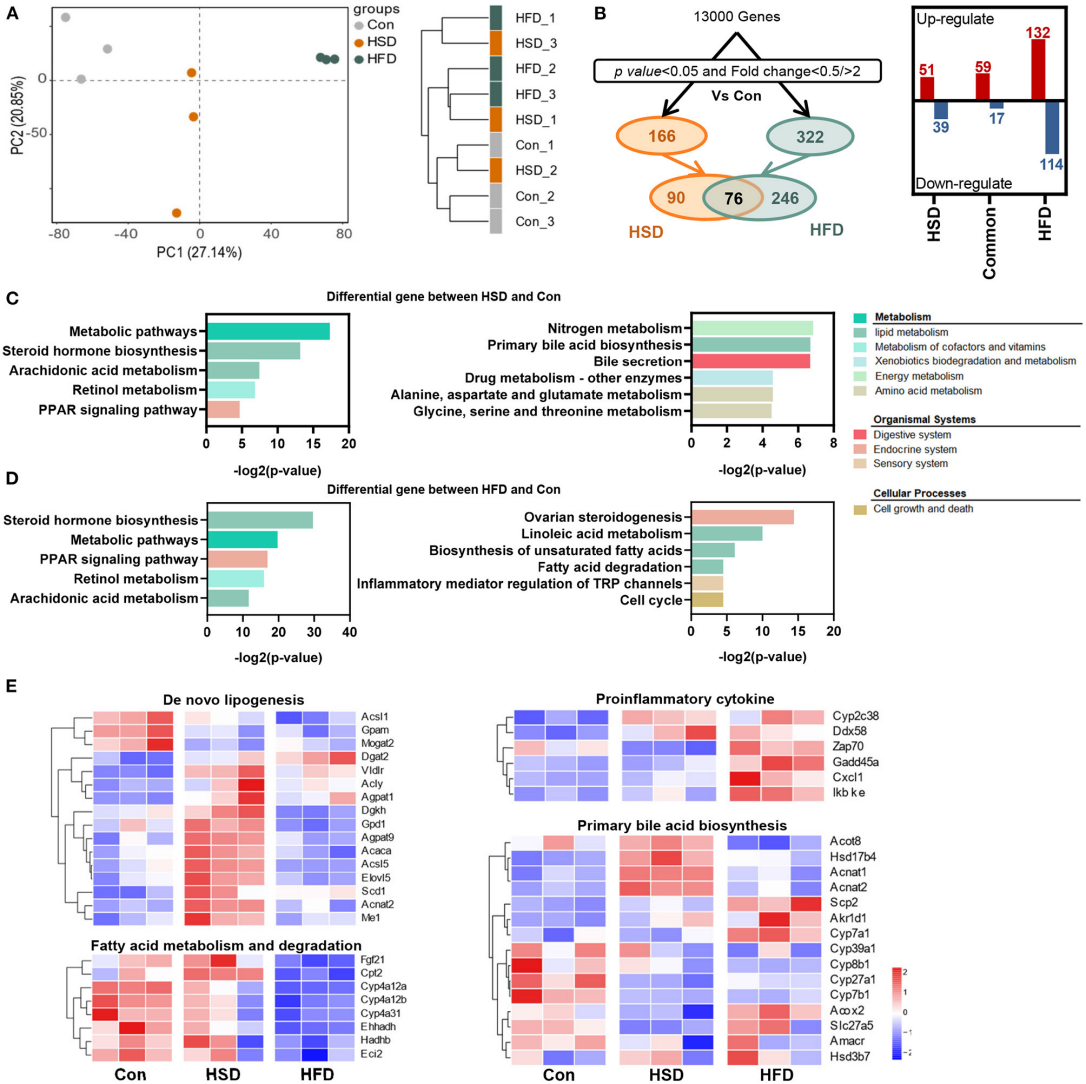
High-fat diet and high-sucrose intake differently altered the hepatic transcriptomic profile. RNA-seq was performed on liver tissues in mice fed with Con, HSD, and HFD for 12 weeks. **(A)** Principal component analysis (PCA) and sample clustering tree among groups. **(B)** Experimental strategy of the genetic screenings. **(C)** KEGG pathway enrichment analysis on the differential gene between HSD and Con. **(D)** KEGG pathway enrichment analysis on the differential gene between HFD and Con. Common pathways were shown on the left, and uniport pathways were shown on the right, in plot D and E. **(E)** Heatmap of genes from lipid metabolism pathway.

To explore the effect of different types of energy intake on liver function, we studied hepatic transcriptome in HFD- and HSD-fed mice after 12-week intervention. First, the principal component analysis (PCA) and sample similarity tree showed a distinct separation on the gene expression profiles of 3 groups (Figure 8A). A total of 166 or 322 differentially expressed genes were determined between Con and HSD and between Con and HFD groups, respectively, with the double criteria of both fold change ≥ 2 (or ≤ 0.5) and *p* < 0.05 (Figure 8B). Interestingly, all 76 common differential genes were observed exactly the same trend in HSD and HFD groups compared with Con group (Figure 8B). We then performed KEGG pathway enrichment analysis on two clusters of differential genes (166 and 322), respectively, using the DAVID Bioinformatics Database (http://david.abcc.ncifcrf.gov/). The results showed that HFD and HSD both significantly altered the metabolic pathway, including lipid metabolism (arachidonic acid metabolism; steroid hormone biosynthesis) and retinol metabolism, along with PPAR signaling pathway in endocrine system (Figures 8C,D). HSD significantly altered the genes mainly related to energy metabolism (nitrogen metabolism), bile acid metabolism (primary bile acid biosynthesis; bile secretion), and amino acid metabolism (glycine, serine, and threonine metabolism; alanine, aspartate, and glutamate metabolism) (Figure 8C). Meanwhile, HFD significantly altered the genes related to ovarian steroidogenesis in endocrine system, lipid metabolism (linoleic acid metabolism; biosynthesis of unsaturated fatty acids; fatty acid degradation), and inflammation (inflammatory mediator regulation of TRP channels) (Figure 8D). Heatmap of enriched genes also showed that HFD significant reduced fatty acid degradation and increased proinflammatory cytokine, while HSD significantly increased hepatic *de novo* lipogenesis and reduced primary bile acid synthesis alternative pathway (Figure 8E).

In summary, different high-energy dietary types have divergent effect on regulating hepatic gene expression. HSD could promote the *de novo* synthesis of fatty acids and reduce primary bile acid synthesis via the alternative pathway in the liver, while HFD intake mainly inhibits the degradation of fatty acids and activates the inflammatory expression in the liver, leading to a severe hepatic lipid accumulation in general.

## Discussion

Metabolic syndrome (MetS) is considered as a pathological state, in which the metabolism of the proteins, fats, carbohydrates, and other substances is disturbed, accompanying a group of complex metabolic disorder syndromes including obesity, hyperlipidemia, hypercholesterolemia, diabetes, and NAFLD (52–54). Increasing evidence showed the significant impact of high-fat and high-sugar diet on the occurrence and development of metabolic syndrome (55, 56). However, very few studies have compared the similarities and differences of metabolic disorders induced by excessive fat and sugar intake, and how different high-energy diet-induced divergent gut microbiota composition and function lead to MetS is still unclear.

In this study, we found that both lipid intake and sugar (sucrose) supplementation were sufficient to induce a pronounced obese phenotype and fatty liver in mice during the 12-week experiment with different extends. Increased visceral fat mass is intimately linked to various metabolic risks. The sharp increase in WAT weight is strongly related to metabolic diseases. Our data showed that HFD induced a fast increase in WAT accumulation at early stage, while HSD played a more persistent effect on increasing WAT during 12-week intervention with higher WAT index in HSD group than HFD group. Interestingly, HSD feeding caused a more significant increase in BAT index, but the increased BAT contains enlarged lipid droplets with pronounced white lipidation, which ultimately leads to mitochondrial dysfunction in BAT (57). We found that 12 weeks of HSD intervention produced hyperlipidemia, manifested by accumulation of serum TG and NEFA, which might be related to the persistent increase in visceral fat weight caused by HSD. Sucrose could be broken into glucose and fructose. On the one hand, glucose could provide a carbon scaffold to promote *de novo* lipogenesis (DNL), which is consistent with the increased expression of DNL-related gene as revealed by our transcriptomic analysis. On the other hand, fructose could turn on the transcriptional program of DNL in the liver and could be metabolized to acetyl-CoA by gut microbiota. In addition, fructose can cause a decrease in lipoprotein lipase, which in turn reduces VLDL level and the clearance of TG-rich lipoproteins, eventually leads to elevated serum TG and NAFA, inducing severe hyperlipidemia (58, 59). In contrast, HFD-induced hypercholesterolemia, impaired glucose tolerance, insulin resistance (IR), and hepatic lipid accumulation were more severe than HSD. Excess dietary fatty acids were taken up by the liver and aggravate liver lipid deposition (60), and ectopic lipid deposition could significantly impair liver function and cause disrupted metabolism of fatty acids. Insulin resistance leads to inappropriate release of fatty acids through dysregulated lipolysis, which further leads to impaired systemic insulin signaling (61), suggesting that HFD-induced lipotoxicity had a greater effect on insulin signaling than sucrose. Meanwhile, liver is the most important organ for cholesterol metabolism. Excessive hepatic cholesterol was transported into the blood through VLDL secretion, resulting in elevated LDL and hypercholesterolemia (62). These findings demonstrate that HFD could induce more severe metabolic disorder than HSD.

In the last two decades, gut microbiota has become an important regulator of host energy metabolism, which is closely related to obesity, NAFLD, and IR (51, 63, 64). Based on 16S rRNA gene sequencing, we found that HFD played a dramatic impact on the structure and function of the gut microbiota throughout the 12-week study. These impacts included significant downregulation of species richness, distinct bacterial community structure from Con group, significant increase in F/B ratio, and dramatic changes in microbial function. We also found that HFD intervention significantly inhibited bacterial glycometabolic-related metabolism pathways and lipid metabolism but activated inflammation signaling. In contrast, there was no significant effect on the overall microbiota structure between HSD and Con group. But after 4-week intervention, the HSD had a significant effect on microbial function such as activation of glycometabolic-related metabolism pathways and inhibition of glycometabolic-related signaling pathway, although the effect was not persistent. Notably, the biosynthesis of primary and secondary bile acids was significantly activated at 12 weeks of HSD intervention, which is consistent with the hepatic transcriptomic results. The above results suggested that the effect of diet on gut microbiota composition and function is time-dependent, and HFD played a more robust and sustained role in regulation gut microbiota than HSD.

The alteration of gut microbiota induced by diet is thought to be closely related to metabolic phenotype (65). Thus, we first analyzed the correlation between mouse phenotype and bacterial family shifted by HSD or HFD, individually. When analyzing HSD vs. Con groups, since HSD induced less bacterial changes, only *Odoribacter* and *Coprococcus_1* were positively correlated, while *Prevotellaceae_NK3B31_group* was negatively associated, with several metabolic indicators. The Spearman correlation based on HFD vs. Con groups showed two separated clusters. Genera elevated by HFD were associated with most mouse phenotypes, and vice versa. *Odoribacter*, the abundance of which was elevated by HFD and late stage of HSD, also showed positive with WAT weight as well as serum and hepatic lipids when comparing HFD vs. Con, but to a less extend when comparing HFD vs. HSD. However, previous studies found *Odoribacter splanchnicus*, a butyric acid-producing bacterium, has a negative correlation with adiposity in pterostilbene-treated Zucker (*fa/fa*) rats (66). In addition, *O. splanchnicus* has been associated with a healthy fasting serum lipid profile in postmenopausal women with obesity (67). The inconsistent findings might be due to the different diet intakes as a standard-chow diet and pterostilbene intervention was used in the rat experiment. Moreover, *Norank_f_Bacteroidales_S24-7_group* and *[Eubacterium]_coprostanoligenes_group*, the abundance of which were reduced by HFD and also showed significant difference between HFD and HSD, showed inverse correlation with mouse metabolic indicators when comparing HFD with Con and HFD with HSD, but not when comparing HSD with Con. This finding indicated that these two genera might account for HFD-induced more severe metabolic disorders than HSD. In addition, based on the different reports of gut microbiota composition after HFD feeding (30–32), the different findings of gut microbiota changes might be due to different mouse model, age, HFD used, and sequencing depth. In addition, metabolomics study might be also important to investigate the potential metabolites that participate in host metabolic regulation. Moreover, the FMT results indicated that phenotypic changes after HFD and HSD feeding were due to the alteration of gut microbiota structure and function to a certain extend. Since the colonization or gut microbiota profile in the recipient mice was not analyzed in this study, the direct relationship between certain bacteria and phenotypes needs to be further investigated.

To further study the mechanism of how the two-kind high-energy diet induce specific host metabolism, we carried out hepatic transcriptomic analysis. As observed, HFD altered more gene changes in numbers. Both HSD and HFD could regulate the pathway of PPAR signaling pathway, retinol metabolism, arachidonic acid metabolism, and steroid hormone biosynthesis. These metabolic pathways have been found to be closely related to metabolic disorders in body: Diabetic patients were frequently accompanied by severe imbalances in vitamin A levels (68); arachidonic acid, as a precursor of various lipid mediators, is involved in inflammatory responses and immune system (69); PPAR signaling pathway had been close related to lipid synthesis and metabolism (70). In addition, HSD significantly inhibits amino acid metabolism pathway and primary bile acid synthesis in alternative pathway. Alanine, serine, threonine, and glycine could generate acetyl coenzyme through pyruvate and then contribute the tricarboxylic acid cycle. The inhibition of their metabolic pathways might be due to the excessive accumulation of acetyl coenzyme A in the liver caused by overdose sugar intake. The alternative pathway for primary bile acid synthesis mainly produced non-12α-hydroxy bile acids. This study suggested that the activation of the alternative pathway can produce beneficial hydroxylated sterols and detoxify harmful hydroxylated sterols, thereby improving metabolic homeostasis (71, 72). Recently, Todoric et al. (73) stated that excessive intake of fructose would cause hepatic de novo lipogenesis. Consistently, after 12 weeks HSD intervention, several important enzymes were significantly upregulated in hepatic for *de novo* fatty acid synthesis, which are more remarkable than HFD. Our results supported that the harmful effect of HSD might be based on the increase in hepatic *de novo* lipogenesis and inhibition of the alternative pathway for primary bile acid synthesis. In contrast, HFD showed significant reduction in fatty acid degradation and increase inflammatory mediator regulation of TRP channels. The widely accepted classical pathogenesis of NAFLD is the “two hit theory,” including the accumulation of lipids in liver cells (the first hit) and inflammatory response triggered by a series of cytotoxic events (the second hit) (74). Triggering of hepatocyte inflammation might be an important reason why HFD produces more severe hepatic metabolic disturbances than HSD.

In conclusion, our results showed that different types of energy intake might have divergent effects on gut microbiota and host metabolism. HSD time-dependently induced metabolic disorder with more severe hypercholesterolemia which might be based on the increase hepatic *de novo* lipogenesis of fatty acid and alternative pathway of bile acid synthesis. In contrast, HFD induced more serious metabolic disorder including obesity, IR, and hepatic lipid accumulation, which might be based on the reduction in fatty acid degradation and increase inflammation. Meanwhile, HFD played a more robust and sustained role than HSD in regulating the structure and function of gut microbiota, and the metabolic detrimental effects can be transferred through fecal microbiota transplantation, suggesting both diet and diet-induced gut microbiota alteration may account for the divergent dietary effect on host metabolism.

## Data Availability Statement

The data presented in the study are deposited in the Data Availability Statement repository, accession number for Transcriptomics Array: GSE206601, GSM6257592, GSM6257593, GSM6257594, GSM6257595, GSM6257596, GSM6257597, GSM6257598, GSM6257599, GSM6257600), for 16S rRNA Gene Sequencing: SRP379710 (PRJNA848196).

## Ethics Statement

The animal study was reviewed and approved by Animal Experiment Institution of Shanghai University of Traditional Chinese Medicine.

## Author Contributions

WZ and YH conducted the FMT animal experiments and data analysis. YuL conducted the time-course animal experiment. YaL, JZ, and XH helped in animal experiments. NZ helped in data analysis. LS designed and revised the manuscript. HL conceptualized and supervised the whole study and revised the manuscript.

## Funding

This work was supported by the Joint Funds of the National Natural Science Foundation of China (No. U21A20413), the National Natural Science Foundation of China (No. 81873059 and 82004016), the Natural Science Foundation of Shanghai (No. 20ZR1453900), Shanghai Excellent Academic Leaders Program (No. 21XD1403500), and Shanghai Pujiang Program (No. 20PJ1413100).

## Conflict of Interest

The authors declare that the research was conducted in the absence of any commercial or financial relationships that could be construed as a potential conflict of interest.

## Publisher's Note

All claims expressed in this article are solely those of the authors and do not necessarily represent those of their affiliated organizations, or those of the publisher, the editors and the reviewers. Any product that may be evaluated in this article, or claim that may be made by its manufacturer, is not guaranteed or endorsed by the publisher.
